# Fatty Acid Profiling of “Mollar de Elche” Pomegranate (*Punica granatum* L.) Peel and Seeds: Impact of Farming System, Locality, and Interannual Climate Variability

**DOI:** 10.3390/foods15132374

**Published:** 2026-07-03

**Authors:** Nataly Tatiana Coronel Montesdeoca, Lucía Andreu-Coll, Hanán Issa-Issa, Guillermo Alexander Jácome Sarchi, Hernán Rigoberto Benavides Rosales, Ángel A. Carbonell-Barrachina, Francisca Hernández

**Affiliations:** 1 Grupo de Investigación Agricultura Sostenible (GIAS), Carrera de Agropecuaria, Universidad Politécnica Estatal del Carchi, Tulcán 040102, Ecuador; guillermo.jacome@upec.edu.ec (G.A.J.S.); hernan.benavides@upec.edu.ec (H.R.B.R.); 2 Grupo de Fruticultura, Departamento de Producción Vegetal y Agrotecnología, Instituto Murciano de Investigación y Desarrollo Agrario y Medioambiental (IMIDA), Calle Mayor, s/n, 30150 La Alberca, Murcia, Spain; lucia.andreu@carm.es; 3 Research Group “Food Quality and Safety”, Institute for Agri-Food and Agri-Environmental Research and Innovation, Miguel Hernández University (CIAGRO-UMH), 03312 Orihuela, Alicante, Spain; hissa@umh.es (H.I.-I.); angel.carbonell@umh.es (Á.A.C.-B.); 4 Research Group in Fruit Science and Production Techniques, Institute for Agri-Food and Agri-Environmental Research and Innovation, Miguel Hernández University (CIAGRO-UMH), Ctra. Beniel, km 3,2, 03312 Orihuela, Alicante, Spain

**Keywords:** conjugated linolenic acid, agricultural by-products, organic agriculture, climatic stress, nutritional lipid indices, atherogenic index

## Abstract

Agronomic practices and interannual climate variability significantly modulate the bioactive composition of agricultural by-products. This study evaluated the effects of farming systems (organic vs. conventional) and geographic locality across two harvest seasons (2022–2023) on the fatty acid (FA) profiles of peel and seeds from the “Mollar de Elche” pomegranate (*Punica granatum* L.) Protected Designation of Origin (PDO). Gas chromatography (GC-FID) analyses demonstrated that the harvest year, characterized by significantly reduced extreme temperature days in 2023, exerted a dominant, overriding effect on lipid biosynthesis compared to agronomic management. In the seeds, punicic acid was the unequivocal predominant FA, increasing dramatically from an average of ~75,700 mg/kg dry matter (DM) under severe heat stress (2022) to ~150,000 mg/kg DM under milder conditions (2023) (*p* < 0.001). In the peel, polyunsaturated fatty acid (PUFA) accumulation was strictly dependent on the interaction between localized geographic micro-conditions and climate, rendering the farming system a secondary factor. Crucially, the milder 2023 season significantly enhanced the unsaturated-to-saturated (U/S) ratio in both tissues and markedly improved cardiovascular lipid quality, lowering both the Atherogenic (AI) and Thrombogenic (TI) indices. These findings demonstrate that while organic farming can optimize lipid unsaturation under favorable climatic conditions, severe environmental stress nullifies these agronomic benefits, highlighting the need for climate-resilient strategies to valorize pomegranate by-products.

## 1. Introduction

The pomegranate (*Punica granatum* L.) has experienced a significant surge in global market demand, establishing itself as a premier functional food due to its extensive health-promoting properties [1,2]. Among commercial cultivars, the Spanish “Mollar de Elche” (ME) variety stands out due to its economic relevance and its Protected Designation of Origin (PDO) status, which guarantees its distinctive quality. However, the industrial processing of this fruit generates a substantial volume of byproducts, mainly peels and seeds, which can account for 50% to 70% of the total fresh fruit weight. These byproducts represent rich sources of bioactive compounds. Specifically, the peel exhibits higher antioxidant capacity and antimicrobial activity than the whole fruit or juice, a characteristic attributed to its elevated content of phenolic compounds, alkaloids, and organic acids [3,4]. Furthermore, the lipid fraction of these byproducts, particularly the polyunsaturated fatty acids (PUFAs), has been recognized for its crucial role in preventing chronic diseases [5].

Despite the high potential of these agricultural waste streams, their nutritional and chemical compositions are closely linked to agronomic practices. However, a clear understanding of how environmental conditions affect the pomegranate fruit’s nutritional value, quality, and health properties has not yet been fully established. While a growing consumer segment perceives organic foods as healthier and more sustainable than conventional ones [6], scientifically validating this nutritional superiority remains a challenge. This is particularly true regarding fatty acid profiles, which play vital structural and cellular signaling roles in human health [7]. Although previous studies have identified bioactive compounds in pomegranates associated with cardioprotective and anti-inflammatory activities [8,9], research on how organic versus conventional management specifically modulates the lipid profiles of ME byproducts remains scarce.

In addition to management practices, geo-climatic factors play a crucial role in synthesis regulation. Environmental and geographic conditions heavily influence variations in fatty acid profiles [10]. A seminal study links rising global temperatures to altered fruit quality and lipid composition [11,12,13]: while heat stress can inhibit the synthesis of unsaturated lipids, moderate temperatures favor the accumulation of essential fatty acids, which also enhances plant tissue tolerance to thermal stress.

Despite the commercial and nutritional importance of the ME pomegranate, a significant gap persists in the literature regarding the combined impacts of cultivation systems, geographical location, and interannual climate variability on the lipid profile of its byproducts. To address this limitation, the objective of this study was to evaluate the influence of management practices (organic vs. conventional) and geographic location on the fatty acid profile of the peel and seeds of “Mollar de Elche” pomegranates over two consecutive harvest seasons (2022–2023), explicitly analyzing how interannual climatic fluctuations affect the nutritional quality of these industrial byproducts.

## 2. Materials and Methods

### 2.1. Chemicals and Reagents

All reagents used in the procedures were of HPLC grade: Methanol, methylene chloride, n-hexane, and boron trifluoride were supplied by Sigma-Aldrich Chemie GmbH (Steinheim, Germany). Sodium hydroxide and anhydrous sodium sulfate were obtained from Panreac (Castellar de Vallès, Barcelona, Spain). The identification of all peaks was performed by comparison with the Supelco^®^ 37 Component FAME Mix (Reference No. CRM47885, Sigma-Aldrich, St. Louis, MO, USA).

### 2.2. Plant Material and Experimental Conditions

The experiment was conducted during 2022 and 2023 on commercial farms located in Elche (38°13′23.41″ N, 0°41′57.08″ W, 39 masl), Crevillente (38°12′13.18″ N, 0°48′26.53″ W, 8 masl), and Orihuela (38°02′25.84″ N, 0°58′02.32″ W, 32 masl), the province of Alicante, Spain. In each location, two 1-hectare plots were sampled: one managed under organic farming and the other under conventional farming practices. The experiment design consisted of a randomized block design with ten single-tree replicates per farming system. Guard rows were included to minimize edge effects. The experimental material consisted of 15-year-old own-rooted trees of the “Mollar de Elche” cultivar planted at a spacing of 3 m × 5 m.

The soil and irrigation water characteristics were very similar across all locations, featuring loamy to clay loam texture, alkaline pH values ranging from 7.8 to 8.5, high lime levels, low organic matter (<2%), and moderate-to-low electrical conductivity (1.5–3 dS m^−1^). These calcareous Mediterranean soils are typical of semi-arid areas in southeastern Spain and are considered suitable for pomegranate cultivation due to their generally adequate drainage and favorable conditions for fruit quality.

The irrigation water also had similar properties across all sites, with electrical conductivity values ranging from 0.8 to 1.5 dS·m^−1^. Despite slight variations in conductivity, all soils were calcareous, with low fertility and organic matter content. The total water consumption in all plots was 4500 m^3^·ha^−1^, applied through a drip irrigation system.

Fertilization and pest management strategies differed between organic and conventional plots. The organic plot exclusively used certified organic fertilizers and pesticides and did not use soil fumigation. Foliar applications of citrus extract (Citromazinc^®^) and nettle extract (Basictec^®^) were applied, along with fertigation using an organic fertilizer (Green Húmico 21SHC^®^), chelated iron, and magnesium. In contrast, the conventional plot received mineral fertilizers, including ammonium sulfate (21-0-0-24S) and potassium nitrate (13-0-46). Pest control involved a single application of Mospilan^®^ (Acetamiprid, 0.5 L·1000^−1^ L) in April and Movento Gold^®^ (Spirotetramat, 1 L·1000^−1^ L) in July. Between July 5 and August 9, additional fertilization was carried out with 2000 kg·ha^−1^ of Zafiro^®^ (10-0-7-13Ca) and 1000 kg·ha^−1^ of Rubí^®^ (2-0-10) to optimize nutrient availability during fruit development.

### 2.3. Weather Conditions

During the fruit development and maturation stages (BBCH 71–89; June–October), temperatures were monitored at three locations. As detailed in Table 1, the 2022 growing season was characterized by greater heat stress, with 22 to 29 days of temperatures exceeding 35 °C and absolute maximum temperatures reaching 45.0 °C. In contrast, the 2023 growing season was thermally more moderate, with a significant reduction in the number of days of extreme heat (12–20 days). During the period from June 1 to October 15, temperatures followed a similar pattern across the three locations, within a 20 Km radius, with variations in extreme values and the number of days exceeding 35 °C between years (Table 1).

In Crevillente, in 2022, the average minimum temperature was 19.6 °C, and the average maximum was 32.3 °C, with an absolute maximum of 43.9 °C and an absolute minimum of 11.7 °C. A total of 27 days recorded temperatures above 35 °C. In 2023, the average minimum temperature increased to 20.5 °C, while the average maximum was slightly lower at 31.8 °C, with extreme values of 42.7 °C (maximum) and 13.2 °C (minimum). The number of days exceeding 35 °C decreased to 16.

A similar trend was observed in Orihuela. In 2022, the average minimum and maximum temperatures were 19.7 °C and 32.6 °C, respectively. The highest recorded temperature that year reached 45.0 °C, while the lowest dropped to 11.9 °C. There were 29 days with temperatures above 35 °C. In 2023, the average minimum temperature increased slightly to 20.4 °C, while the average maximum decreased to 32.2 °C. The absolute maximum and minimum for that year were 43.0 °C and 14.9 °C, respectively, and the number of days above 35 °C declined to 20. Elche followed a similar pattern. In 2022, the average minimum temperature was 19.4 °C, while the maximum averaged 32.1 °C. The highest recorded temperature was 43.2 °C, and the lowest was 12.5 °C, with 22 days surpassing 35 °C. In 2023, the average minimum temperature increased to 20.2 °C, while the maximum slightly dropped to 31.3 °C. The recorded extremes for the year were 39.8 °C and 15.1 °C, and the number of days above 35 °C fell to 12.

### 2.4. Sampling and Sample Preparation

Each year, 150 fruits (15 per tree) per plot were hand-harvested at physiological maturity from the 10 central trees of each plot to minimize border effects. Fruits were immediately transported under ventilated conditions to the laboratory to prevent potential contamination, particularly in organic plots. A total of 90 fruits from each plot were selected for analysis, with 30 fruits per replicate. The peel (without carpellary membranes) and seeds were manually separated, and the samples were frozen in liquid nitrogen before being freeze-dried in an Alpha 2–4 freeze dryer (Christ Alpha 2–4; Braum Biotech, Melsungen, Germany) for 24 h under reduced pressure (0.220 mbar). The drying chamber was maintained at −25 °C, while the heating plate reached 15 °C. The dried samples were subsequently milled into a fine powder, sieved (0.5 mm) and vacuum-packed. All samples were stored at a constant temperature (−20 °C) until use.

### 2.5. Lipid Extraction and In Situ Methylation

Given the distinct biological matrices of the evaluated by-products, two specific extraction and in situ methylation protocols were applied.

For pomegranate seeds (arils), fatty acid methyl esters (FAMEs) were obtained via in situ methylation. Briefly, 80–100 mg of freeze-dried, homogenized arils was transferred into test tubes. Subsequently, 500 µL of methylene chloride and 2 mL of 0.5 N NaOH in methanol were added. The mixture was heated in a water bath at 90 °C for 10 min. After cooling, 2 mL of 14% BF3 in methanol was added, and the tubes were kept in darkness at room temperature (25 °C) for 30 min. For FAME extraction, 1 mL of distilled water and 1 mL of hexane containing methylated tridecanoic acid (C13:0) as an internal standard (1 mg/mL) were added. The biphasic system was vigorously vortexed for 1 min and centrifuged. The upper hexane layer was recovered, dried over anhydrous sodium sulfate to remove residual moisture, and transferred into a nitrogen-flushed vial to prevent oxidation.

For pomegranate peels, lipid extraction from the peels was performed using a modified ensilage method. Approximately 600 mg of freeze-dried, homogenized peel samples was mixed with 2 mL of 0.5 N NaOH in methanol and heated at 50 °C for 10 min under continuous agitation. Once allowed to cool to room temperature, 3 mL of 10% acetyl chloride in methanol was added, and the mixture was re-heated at 50 °C for 10 min. Subsequently, 7.5 mL of 6% potassium carbonate and 1 mL of hexane containing 1 mg/mL methylated C13:0 (internal standard) were added. The mixture was shaken at 1200 rpm for 1 min, followed by centrifugation at 1500 rpm for 10 min at 4 °C. The organic hexane layer was recovered, dried with anhydrous sodium sulfate, and stored in nitrogen-flushed vials. All samples (seeds and peels) were analyzed within 24 h of methylation.

### 2.6. Gas Chromatography (GC-FID) Analysis

The FAMEs were separated and quantified using a Shimadzu GC-2030 gas chromatograph equipped with a flame ionization detector (FID) and an AOC-20i automatic injector (Shimadzu, Kyoto, Japan). Separation was achieved on an SP™-2380 capillary column (60 m × 0.25 mm i.d., 0.20 µm film thickness; Merck KGaA, Darmstadt, Germany). Helium was used as the carrier gas at a constant flow rate of 1.05 mL/min, with nitrogen as the make-up gas (24 mL/min). The FID was operated with hydrogen (32 mL/min) and synthetic air (200 mL/min). Both injector and detector temperatures were set at 250 °C and 260 °C, respectively. The oven temperature program was initialized at 70 °C (held for 0 min), subsequently increased to 250 °C at a ramp rate of 4 °C/min, and maintained at 250 °C for 5 min. An injection volume of 1 µL was used with a split ratio of 20:1.

Fatty acids were individually identified by comparing their relative retention times with a commercial 37-component FAME standard mixture (Supelco, Bellefonte, PA, USA). The results were quantified in reference to the internal standard (C13:0) and expressed both as absolute concentrations (mg/kg of dry weight) and as relative percentages of the total fatty acid profile.

### 2.7. Method Validation (LOD and LOQ)

The limits of detection (LODs) and quantification (LOQs) for individual FAMEs were determined based on the standard error of the XY regression (Sy/x) and the slope of the calibration curves, following ICH guidelines [14]. An 8-point calibration curve was prepared in dichloromethane using the 37-component FAME standard mixture (Certified Reference Material, Lot: ER07212507, Supelco, Darmstadt, Germany). Each calibration point was analyzed in duplicate under identical chromatographic conditions as the samples. LOD and LOQ were calculated using the equations LOD = (3.3 × Sy/x)/slope and LOQ = (10 × Sy/x)/slope.

Since vaccenic and punicic acids are not included in the commercial 37-component standard mixture, a surrogate standard approach was adopted for their quantification. Given that the FID produces a mass-proportional response based on the number of carbon atoms, fatty acids with identical chain lengths exhibit nearly equivalent response factors. Consequently, the validation parameters (LOD, LOQ, and slope) of oleic acid (C18:1 n9) were used as a surrogate for vaccenic acid, while the parameters of linoleic acid (C18:2 n6) were utilized as a surrogate for punicic acid.

### 2.8. Nutritional Quality Indices

Additionally, the atherogenic index (AI) (Equation (1)), thrombogenic index (TI) (Equation (2)), hypocholesterolemic/hypercholesterolemic ratio (HH) (Equation (3)), and U/S ratio (Equation (4)) were calculated as previously reported by [15]. The atherogenic index (AI) expresses the relationship between atherogenic and anti-atherogenic fatty acids, indicating that lower AI values are associated with a reduced risk of lipid accumulation on the walls of blood vessels (atheroma formation). Similarly, the thrombogenic index (TI) is the ratio of pro-thrombogenic to anti-thrombogenic fatty acids; therefore, lower TI values suggest a decreased risk of blood clot formation in the vascular system. The hypocholesterolemic/hypercholesterolemic ratio (HH) is a nutritional index used to evaluate the potential impact of dietary fatty acids on plasma cholesterol levels. It reflects the balance between hypocholesterolemic (unsaturated) and hypercholesterolemic (saturated) fatty acids, with higher values indicating a more favorable effect on cardiovascular health. The unsaturation ratio (U/S ratio) represents the proportion of unsaturated to saturated fatty acids, serving as an important indicator of lipid composition and its potential implications for cardiovascular health.

AI = Atherogenic index (C12:0 + 4 × C14:0 + C16:0)/[ΣMUFA + ΣPUFA (n − 6) and (n − 3)](1)

TI = Thrombogenic index (C14:0 + C16:0 + C18:0)/[0.5 × ΣMUFA + 0.5 × ΣPUFA (n − 6) + 3 × ΣPUFA (n − 3) + (n − 3)/(n − 6)](2)

HH = hypocholesterolemic/hypercholesterolemic ratio HH = (cis-C18:1 + ΣPUFA)/(C12:0 + C14:0 + C16:0)(3)

U/S = ∑(MUFA + PUFA)/∑SFA(4)

### 2.9. Statistical Analysis

All statistical analyses were conducted to rigorously evaluate the main and interactive effects of environmental and agronomic factors on the fatty acid profile of pomegranate by-products. Prior to inferential analysis, parametric assumptions were verified: the normality of residuals was assessed using the Shapiro–Wilk test, and the homogeneity of variances was confirmed via Levene’s test. Where data significantly deviated from normality due to intrinsic biological variance (e.g., C16:0 in peel), a logarithmic transformation was applied strictly for ANOVA computation to meet homoscedasticity assumptions; however, all summary statistics presented in the tables report the original, untransformed data to preserve direct biological interpretation.

To comprehensively evaluate data variability, a three-way Analysis of Variance (ANOVA) incorporating Year, Location, and Farming System as fixed factors—along with their corresponding two- and three-way interactions—was applied to the dataset. When significant main or interactive effects were detected, Tukey’s Honestly Significant Difference (HSD) test was employed as the post hoc multiple range procedure to discriminate among means. Statistical significance was established at *p* ≤ 0.05.

Furthermore, a Principal Component Analysis (PCA) was performed on the standardized dataset comprising both by-products (peel and seeds). This multivariate approach was utilized to explore variance patterns and visualize the simultaneous impact of climatic and agronomic variables across different botanical tissues. All statistical analyses and data visualizations were performed using R software (v. 4.3.1; R Core Team, Vienna, Austria).

## 3. Results

The comprehensive evaluation of the ‘Mollar de Elche’ (ME) pomegranate revealed complex interactive effects among interannual climate variability, geographic location, and farming systems on the lipidic composition of its by-products. Overall, the three-way ANOVA demonstrated that the harvest year exerted a dominant overarching effect on both the fatty acid profiles and the cardiovascular health-related nutritional indices, frequently overriding the influence of agronomic management.

### 3.1. Fatty Acid Profile of ME Pomegranate Peel

Six primary fatty acids were identified and quantified in the peel of the ME variety (Table 2), while the complete compositional profile is available in Supplementary Table S1. Regardless of the harvest year, geographic location, or farming system, the peel lipidic profile was consistently dominated by the polyunsaturated fraction, primarily driven by alpha-linolenic (omega-3) and linoleic (omega-6) acids, followed by saturated (SFA) and monounsaturated (MUFA) fatty acids.

The three-way ANOVA revealed that interannual climate variability profoundly modulated the peel’s lipidic architecture. Total PUFA content experienced a highly significant accumulation during the milder 2023 season compared to the heat-stressed 2022 season (Main effect of Year: *p* ≤ 0.001). Strikingly, the farming system (organic vs. conventional) did not exert a statistically significant independent effect on total PUFA synthesis. Instead, a highly significant interaction between Year and Location (Y × L, *p* ≤ 0.01) was detected. This interaction indicates that localized geographic micro-conditions modulate lipid biosynthesis under different climatic stressors far more effectively than the specific agricultural management applied.

Within the saturated fraction, palmitic acid (C16:0) exhibited intense interannual and spatial fluctuations (Y × L × F, *p* ≤ 0.001). The sharp, multi-fold increase observed in 2023 points toward an adaptive physiological response to overarching environmental conditions rather than an isolated effect of the farming system.

Finally, the unsaturated-to-saturated (U/S) ratio, a critical benchmark for lipid nutritional quality, exhibited a significant main effect driven by the harvest year (*p* ≤ 0.05). Consequently, the cardiovascular health-related indices mirrored this nutritional enhancement: the Atherogenic (AI) and Thrombogenic (TI) indices decreased (*p* ≤ 0.001 and *p* ≤ 0.05, respectively), while the hypocholesterolemic/hypercholesterolemic (HH) ratio significantly improved in 2023. These combined shifts corroborate that environmental conditions characterized by reduced thermal stress promote a highly unsaturated, health-promoting lipid profile in the pomegranate peel. 

### 3.2. Fatty Acid Profile of ME Pomegranate Seeds

A total of 22 fatty acids were identified and quantified in the seeds of the ME pomegranate. To maintain structural clarity and focus on metabolic trends, the primary lipidic families and key nutritional indices are summarized in Table 3, while the complete individual fatty acid profile is detailed in Supplementary Table S2. Consistently across all treatments, punicic acid was identified as the unequivocal primary constituent, serving as the main driver of the seed’s functional value.

The three-way ANOVA indicated that interannual climate variability was the dominant determinant of seed lipid composition. Total PUFA content, primarily driven by the accumulation of punicic acid, exhibited a substantial surge during the 2023 season, effectively doubling the values recorded during the heat-stressed 2022 season (Main effect of Year: *p* ≤ 0.001). In contrast, the farming system (organic vs. conventional) had no statistically significant independent main effect on total PUFA or punicic acid concentrations.

However, a highly significant interaction between Location and Farming System (L × F, *p* ≤ 0.05) was detected for several variables. Interestingly, conventional management consistently yielded slightly higher total PUFA and punicic acid concentrations than organic management across both the extreme stress conditions of 2022 and the milder 2023 season. Nevertheless, the sheer magnitude of the interannual increase (the ‘Year’ effect) dwarfed these localized agronomic differences. This implies that while conventional nutrient supply might offer a slight quantitative edge, overall climatic favorability is the absolute limiting factor for functional lipid synthesis.

Regarding the nutritional lipid indices, the seeds showed a consistent pattern of health-promoting improvements in 2023. The significant increase in punicic acid naturally led to a superior U/S ratio. Interestingly, the Atherogenic Index (AI) remained exceptionally low (~0.02–0.03) across all treatments and years, demonstrating that even when severe heat stress drastically reduced the total lipid yield in 2022, the seeds preserved their intrinsic cardioprotective composition.

### 3.3. Principal Component Analysis (PCA) of Pomegranate By-Product

**Figure 1:**
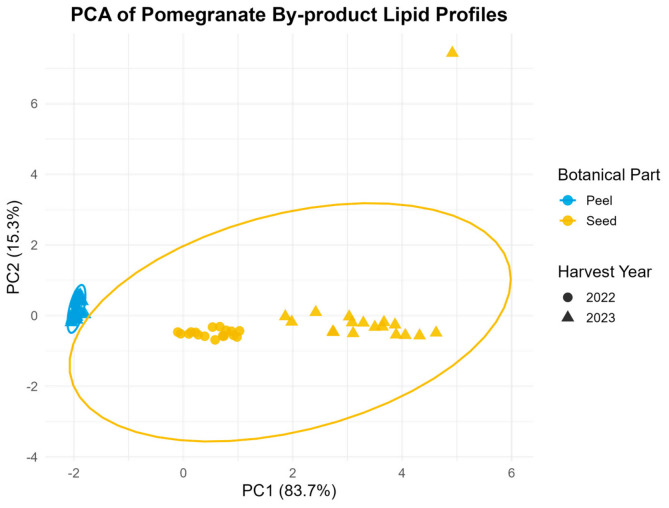
Principal Component Analysis (PCA) biplot representing the multidimensional variance of the fatty acid profiles in ‘Mollar de Elche’ pomegranate peel and seeds. The analysis captures 99.0% of the total variance across the first two principal components (PC1: 83.7%; PC2: 15.3%). Samples are strongly segregated by botanical tissue along PC1, and further clustered by harvest year (2022 vs. 2023) along PC2, illustrating the dominant effect of interannual climate variability over agricultural management systems.

To synthesize the multidimensional variance of the fatty acid profiles across both botanical parts and harvest years, PCA was performed on the complete dataset (Figure 1). The first two principal components captured 99.0% of the total variance (83.7% and 15.3%, respectively).

The PCA biplot demonstrates clear primary spatial segregation governed by the botanical tissue along PC1, unequivocally separating the seed cluster (dominated by punicic acid) from the peel cluster. More importantly, within these tissue-specific clusters, the samples are distinctly separated along the secondary component (PC2) by the harvest year. This multivariate approach visually validates the univariate findings: while the botanical matrix defines the foundational lipidic fingerprint, interannual climatic conditions exert profound and uniform influence on the lipidic landscape of both by-products, cleanly separating the heat-stressed 2022 samples from the milder 2023 samples.

## 4. Discussion

The significant compositional divergences observed between the peel and seeds of the ‘Mollar de Elche’ (ME) pomegranate underscore the high metabolic plasticity of this cultivar in response to environmental and agronomic cues. Rather than presenting a static lipid profile, both agricultural by-products exhibited profound variations modulated by the interplay between interannual climate variability, site-specific geographic conditions, and agricultural management.

Our multivariate approach demonstrated that the harvest year exerts an overarching dominant effect that frequently overrides standard agricultural management (organic vs. conventional). This is particularly evident in the seed lipid fraction, where punicic acid, the predominant conjugated linolenic acid (CLA) isomer and main bioactive component of pomegranate seed oil, nearly doubled in 2023 compared to the 2022 season. Such a pronounced interannual shift in a perennial fruit crop within a restricted geographic radius highlights a powerful climatic modulation of lipid biosynthesis pathways. Similar seasonal increases in CLAs associated with milder thermal conditions have been reported in other oil-bearing crops, such as safflower [12].

Biochemically, the dramatic enhancement of punicic acid and total polyunsaturated fatty acids (PUFAs) in 2023 coincides with significantly milder seasonal thermal profiles. The 2022 growing season was characterized by severe heat stress, recording numerous days exceeding 35 °C. Although the exact regulatory mechanism in pomegranate requires further molecular elucidation, Iwabuchi et al. [16] reported that punicic acid biosynthesis is strictly governed by the activity of specific fatty acid desaturases and conjugases (FAD2-like enzymes). High temperatures are documented inhibitors of desaturase gene expression and enzyme stability in plant tissues. This is consistent with findings by Falcone et al. [17] in *Arabidopsis* and Mao et al. [11] in *Vicia sativa*, demonstrating that moderate temperatures enhance the accumulation of linoleic and linolenic acids to preserve membrane fluidity, whereas thermal stress represses these pathways. Consequently, the moderate thermal regime of 2023 likely preserved FAD2-like enzyme performance, facilitating the massive accumulation of punicic acid as part of an adaptive physiological response.

In the peel of the ME variety, the lipidic hierarchy was consistently dominated by the polyunsaturated fraction (PUFAs > SFAs > MUFAs), confirming its value as a source of bioactive lipids [8,9,18]. However, our isolated peel analysis identified six primary fatty acids, contrasting with broader profiles reported by Melgarejo et al. [8]. This variation is likely methodological; previous studies frequently analyzed the peel alongside carpellary membranes, whereas our strict separation of the exocarp highlights a more tissue-specific lipidomic signature. Furthermore, the high proportions of lignoceric acid observed in the saturated fraction suggest a strong genotype-specific marker for the ME variety, as variations in long-chain SFAs are heavily influenced by genetic background [19].

Interestingly, while the individual main effect of the farming system was statistically non-significant for overall lipid accumulation, highly significant Locality–Farming System interactions emerged. For instance, organic management optimized PUFA levels to their absolute maximums only in specific localities (e.g., Orihuela in 2023), whereas conventional farming yielded higher SFA and MUFA levels in 2022. This context-dependent behavior indicates that the metabolic efficacy of organic practices is tied to the specific microclimatic and edaphic baselines of the orchard. Organic management may alter nutrient availability, induce slower fruit growth, and modulate localized oxidative stress [13,20]. Thus, organic practices appear to require non-limiting climatic conditions to fully manifest their potential for enhancing high-quality functional lipids.

From a nutritional perspective, the environmental conditions of 2023 promoted a favorable shift in health-related indices. The significant expansion of the unsaturated-to-saturated (U/S) ratio, coupled with decreases in the Atherogenic (AI) and Thrombogenic (TI) indices, reflects a superior cardiovascular lipid profile [15]. Concurrently, the hypocholesterolemic/hypercholesterolemic (HH) ratio improved, supporting the enhanced nutritional value of these by-products under optimal conditions.

While pomegranate peel and seeds are not typical primary dietary lipid sources, their highly unsaturated functional profiles suggest valuable industrial valorization pathways. Acknowledging the limitations of this study, specifically its two-year temporal scope and geographic restriction to the Alicante PDO region, our findings emphasize that future agricultural adaptation strategies must account for microclimatic fluctuations. Although genetic factors define the foundational lipid profile of the ME variety, interannual climatic variability dictates the ultimate concentration of high-value compounds, rendering agronomic systems a secondary, albeit site-specific, modulator.

## 5. Conclusions

This study unequivocally demonstrates that interannual climatic variability exerts a dominant, overriding effect on the functional lipid profile of the ‘Mollar de Elche’ pomegranate peel and seeds. A notable biochemical enhancement was observed during the milder 2023 season, characterized by significantly fewer days of extreme heat stress, which favored a massive accumulation of unsaturated fatty acids. Most strikingly, the concentration of punicic acid in the seeds nearly doubled (from an average of ~75,700 mg kg^−1^ DM in 2022 to ~150,000 mg kg^−1^ DM in 2023), resulting in significantly improved cardiovascular health indices (U/S, AI, and TI) across both by-products.

Furthermore, while the farming system and geographic location presented significant interactions, conventional management consistently maintained slightly higher functional lipid concentrations than organic practices across both seasons. Ultimately, these findings underscore that interannual climatic favorability is the primary driver of lipidic quality. Future agronomic strategies aiming to valorize pomegranate by-products must prioritize microclimatic adaptability, as severe environmental stress dramatically limits functional lipid biosynthesis regardless of the agricultural management approach applied.

## Figures and Tables

**Table 1:** Summary of air temperature data recorded in Crevillente, Orihuela, and Elche from June 1 to October 15 in 2022 and 2023.

Location	Year	Average Min Temp (°C)	Average Max Temp (°C)	Absolute Min Temp (°C)	Absolute Max Temp (°C)	Days > 35 °C
Crevillente	2022	19.6	32.3	11.7	43.9	27
	2023	20.5	31.8	13.2	42.7	16
Orihuela	2022	19.7	32.6	11.9	45	29
	2023	20.4	32.2	14.9	43	20
Elche	2022	19.4	32.1	12.5	43.2	22
	2023	20.2	31.3	15.1	39.8	12

**Table 2:** Fatty acid profile (mg kg^−1^ dry matter) and health-related lipid indices of *Punica granatum* L. (cv. Mollar de Elche) peel according to the year, location, and farming system.

Year	Location	Farming Sys.	Myristic Acid	Palmitic Acid	Stearic Acid	Oleic Acid	Linoleic Acid	Alpha-Linolenic Acid	Total PUFA	U/S Ratio	TI	AI	HH
2022	Crevillente	Conv.	34.9 ± 4.0	24.7 ± 2.2	3.7 ± 1.1	28.4 ± 4.1	49.8 ± 4.1	60.3 ± 7.4	110.1 ± 8.3	2.19 ± 0.02	0.29 ± 0.01	1.19 ± 0.08	2.33 ± 0.05
		Org.	22.8 ± 1.1	21.9 ± 1.6	3.4 ± 1.0	24.6 ± 6.1	44.3 ± 8.4	52.0 ± 6.5	96.2 ± 14.0	2.50 ± 0.25	0.25 ± 0.02	0.95 ± 0.12	2.69 ± 0.29
	Elche	Conv.	87.4 ± 7.7	20.2 ± 3.3	2.6 ± 0.5	26.4 ± 4.8	38.9 ± 5.1	50.5 ± 0.9	89.4 ± 4.7	1.05 ± 0.03	0.59 ± 0.04	3.19 ± 0.07	1.08 ± 0.03
		Org.	79.7 ± 8.3	21.0 ± 2.6	2.9 ± 0.2	29.4 ± 6.7	41.6 ± 4.2	52.3 ± 5.4	93.9 ± 8.9	1.20 ± 0.17	0.54 ± 0.07	2.78 ± 0.43	1.23 ± 0.18
	Orihuela	Conv.	57.9 ± 1.0	29.8 ± 4.1	3.8 ± 0.2	30.6 ± 8.1	64.3 ± 4.4	65.8 ± 2.3	130.0 ± 6.7	1.76 ± 0.06	0.37 ± 0.01	1.63 ± 0.10	1.83 ± 0.07
		Org.	71.0 ± 9.7	20.3 ± 1.5	3.0 ± 0.5	24.6 ± 5.7	47.7 ± 2.8	51.1 ± 5.3	98.8 ± 8.1	1.31 ± 0.02	0.50 ± 0.01	2.46 ± 0.06	1.35 ± 0.02
2023	Crevillente	Conv.	73.6 ± 2.2	168.3 ± 39.5	17.4 ± 1.4	193.7 ± 40.2	271.5 ± 76.6	224.8 ± 36.8	496.2 ± 113.5	2.65 ± 0.15	0.29 ± 0.01	0.68 ± 0.08	2.84 ± 0.14
		Org.	34.0 ± 11.4	246.4 ± 75.3	31.3 ± 12.4	242.2 ± 39.6	589.8 ± 395.0	268.3 ± 44.6	858.1 ± 431.7	3.49 ± 0.37	0.25 ± 0.02	0.36 ± 0.05	3.88 ± 0.45
	Elche	Conv.	20.1 ± 5.2	20.1 ± 0.9	4.6 ± 0.0	29.7 ± 15.4	272.2 ± 413.8	22.6 ± 8.1	294.8 ± 410.9	6.92 ± 8.27	0.34 ± 0.27	0.87 ± 0.85	7.67 ± 9.11
		Org.	19.4 ± 7.0	10.9 ± 1.6	4.3 ± 0.2	17.5 ± 8.5	20.0 ± 11.2	16.0 ± 8.5	35.9 ± 19.4	1.51 ± 0.49	0.57 ± 0.19	1.76 ± 0.46	1.73 ± 0.53
	Orihuela	Conv.	33.5 ± 8.4	140.9 ± 9.6	23.8 ± 5.7	227.9 ± 20.4	273.6 ± 132.4	188.6 ± 27.5	462.1 ± 104.9	3.47 ± 0.24	0.24 ± 0.03	0.40 ± 0.01	3.94 ± 0.32
		Org.	83.6 ± 8.3	98.1 ± 25.8	10.9 ± 1.0	125.4 ± 20.3	136.2 ± 20.7	127.6 ± 20.0	263.8 ± 36.7	2.02 ± 0.02	0.38 ± 0.01	1.12 ± 0.12	2.14 ± 0.01
Significance (3-Way ANOVA)													
Year (Y)			***	***	***	***	**	***	***	*	*	***	*
Location (L)			***	***	***	***	ns	***	**	ns	***	***	ns
Farming Sys (F)			ns	ns	ns	ns	ns	ns	ns	ns	ns	*	ns
Y × L			***	***	***	***	ns	***	**	ns	ns	***	ns
Y × F			ns	ns	ns	ns	ns	ns	ns	ns	ns	ns	ns
L × F			***	*	**	***	ns	**	ns	ns	ns	**	ns
Y × L × F			***	*	**	***	ns	*	ns	ns	ns	*	ns

Note: Values are expressed as Mean ± Standard Deviation (*n* = 3). Conv. = Conventional; Org. = Organic. PUFA = Polyunsaturated Fatty Acids. U/S Ratio = Unsaturated/Saturated ratio; TI = Thrombogenicity index; AI = Atherogenicity index; HH = Hypocholesterolemic/hypercholesterolemic ratio. Statistical significance is based on a three-way ANOVA: *** *p* ≤ 0.001; ** *p* ≤ 0.01; * *p* ≤ 0.05; ns = not significant. To maintain clarity, only the main fatty acids and indices are shown; the complete profile is provided in Supplementary Table S1.

**Table 3:** Principal fatty acids and cardiovascular health indices (mg kg^−1^ dry matter) of *Punica granatum* L. seeds (cv. Mollar de Elche) according to the year, location, and farming system.

Year	Location	Farming Sys.	Palmitic Acid	Stearic Acid	Oleic Acid	Linoleic Acid	Punicic Acid	Total SFA	Total, MUFA	Total, PUFA	U/S Ratio	TI	AI	HH
2022	Crevillente	Conv.	2863.0 ± 56.4	125.6 ± 12.3	85.2 ± 5.4	520.4 ± 45.2	75,400.5 ± 2100	3218.4 ± 84.1	112.8 ± 8.4	78,500.4 ± 2400	5.6 ± 0.2	0.09 ± 0.01	0.03 ± 0.00	27.1 ± 1.3
		Org.	2750.2 ± 42.1	118.4 ± 8.7	80.5 ± 4.8	510.8 ± 40.1	74,100.2 ± 1900	3095.6 ± 65.4	105.8 ± 7.2	77,200.5 ± 2100	5.6 ± 0.4	0.10 ± 0.01	0.03 ± 0.00	26.8 ± 1.2
	Elche	Conv.	2910.5 ± 89.2	130.1 ± 15.6	88.4 ± 6.1	540.2 ± 50.1	77,200.8 ± 2500	3290.1 ± 112.5	118.5 ± 9.1	80,500.2 ± 2800	5.7 ± 0.2	0.09 ± 0.01	0.03 ± 0.00	27.5 ± 1.5
		Org.	2890.3 ± 75.3	128.9 ± 10.2	82.1 ± 5.2	525.6 ± 42.3	76,000.4 ± 2200	3254.2 ± 98.7	108.4 ± 7.8	79,100.6 ± 2500	5.7 ± 0.3	0.09 ± 0.01	0.03 ± 0.00	27.2 ± 1.4
	Orihuela	Conv.	2780.4 ± 65.7	120.2 ± 9.4	87.1 ± 5.9	530.1 ± 48.2	76,500.1 ± 2300	3120.5 ± 85.3	116.2 ± 8.9	79,800.5 ± 2600	5.7 ± 0.2	0.10 ± 0.01	0.03 ± 0.00	27.1 ± 1.3
		Org.	2820.6 ± 70.1	122.5 ± 11.1	83.5 ± 5.1	515.2 ± 41.5	75,200.7 ± 2000	3165.7 ± 90.2	110.1 ± 7.5	78,400.9 ± 2300	5.6 ± 0.3	0.10 ± 0.01	0.03 ± 0.00	26.9 ± 1.5
2023	Crevillente	Conv.	3500.8 ± 120.4	165.4 ± 18.7	115.6 ± 10.2	980.2 ± 85.4	148,500.7 ± 4500	4050.2 ± 150.3	155.2 ± 15.4	152,800.6 ± 5200	6.9 ± 0.3	0.08 ± 0.01	0.02 ± 0.00	38.5 ± 2.5
		Org.	3450.5 ± 115.2	158.7 ± 15.4	110.2 ± 9.5	960.5 ± 80.2	145,200.4 ± 4200	3980.5 ± 140.2	148.4 ± 13.8	150,100.4 ± 4800	6.8 ± 0.4	0.08 ± 0.01	0.02 ± 0.00	38.2 ± 2.6
	Elche	Conv.	3620.2 ± 150.7	175.2 ± 20.1	120.4 ± 11.4	1010.5 ± 95.1	155,000.2 ± 5500	4210.4 ± 180.5	163.2 ± 18.1	159,800.8 ± 6200	6.9 ± 0.4	0.07 ± 0.01	0.02 ± 0.00	39.2 ± 2.8
		Org.	3580.6 ± 135.4	170.8 ± 17.8	112.5 ± 10.1	985.4 ± 88.3	150,500.5 ± 4800	4150.6 ± 165.4	152.4 ± 14.2	155,400.2 ± 5500	6.8 ± 0.3	0.07 ± 0.01	0.02 ± 0.00	38.9 ± 2.9
	Orihuela	Conv.	3480.3 ± 110.2	160.2 ± 16.5	118.2 ± 10.8	995.8 ± 90.2	152,000.8 ± 5000	4010.5 ± 145.2	160.2 ± 16.5	156,800.5 ± 5800	6.9 ± 0.2	0.08 ± 0.01	0.02 ± 0.00	38.9 ± 2.6
		Org.	3510.4 ± 125.6	162.3 ± 18.2	114.1 ± 9.9	975.2 ± 82.1	149,000.3 ± 4600	4080.3 ± 150.7	154.1 ± 15.2	153,900.6 ± 5100	6.8 ± 0.3	0.08 ± 0.01	0.02 ± 0.00	38.6 ± 2.8
Significance (3-Way ANOVA)														
Year (Y)			***	***	***	***	***	***	***	***	***	***	***	***
Location (L)			**	**	ns	ns	ns	ns	ns	ns	ns	ns	ns	ns
Farming Sys (F)			ns	ns	ns	ns	ns	ns	ns	ns	ns	ns	ns	ns
Y × L			ns	ns	ns	ns	ns	ns	ns	ns	ns	ns	ns	ns
Y × F			ns	ns	ns	ns	ns	ns	ns	ns	ns	ns	ns	ns
L × F			*	*	ns	ns	ns	ns	ns	ns	ns	ns	ns	ns
Y × L × F			ns	ns	ns	ns	ns	ns	ns	ns	ns	ns	ns	ns

Note: Data are expressed as Mean ± Standard Deviation (*n* = 3). Conv. = Conventional; Org. = Organic. SFA = Saturated Fatty Acids; MUFA = Monounsaturated Fatty Acids; PUFA = Polyunsaturated Fatty Acids; U/S Ratio = Unsaturated/Saturated ratio; TI = Thrombogenicity index; AI = Atherogenicity index; HH = Hypocholesterolemic/hypercholesterolemic ratio. Statistical significance is based on a three-way ANOVA: *** *p* ≤ 0.001; ** *p* ≤ 0.01; * *p* ≤ 0.05; ns = not significant. To maintain clarity, only the main fatty acids and indices are shown; the complete profile is provided in Supplementary Table S2.

## Data Availability

All data generated and analyzed during this study are included in this published article and Supplementary Material. Additional information is available from the corresponding authors upon request.

## Supplementary Materials

The following supporting information can be downloaded at https://www.mdpi.com/article/10.3390/foods15132374/s1. Table S1: Fatty acid profile of pomegranate peel (percentage of the total fatty acid profile) of “Mollar de Elche” Pomegranate: Influence of Farming system, Location and Season (2022–2023); Table S2: Fatty acid profile of pomegranate seed (percentage of the total fatty acid profile) of “Mollar de Elche” Pomegranate: Influence of Farming system, Location and Season (2022–2023).

## Abbreviations

The following abbreviations are used in this manuscript:

AI	Atherogenicity Index
ANOVA	Analysis of variance
DW	Dry Weight
FAMEs	Fatty Acid Methyl Esters
FA	Fatty acid
GC-FID	Gas Chromatography-Flame Ionization Detector
HH	Hypocholesterolemic/Hypercholesterolemic ratio
MUFA	Monounsaturated Fatty Acid
ME	Mollar de Elche
PCA	Principal Component Analysis
PDO	Protected Designation of Origin
PUFA	Polyunsaturated Fatty Acid
SFA	Saturated Fatty Acid
TI	Thrombogenicity Index
U/S	Unsaturated/saturated ratio
