# Supplementary material for: Fatty Acid Profiling of “Mollar de Elche” Pomegranate (Punica granatum L.) Peel and Seeds: Impact of Farming System, Locality, and Interannual Climate Variability
Source: Foods. 2026 Jul 3;15(13):2374. doi: 10.3390/foods15132374 (PMC13362359; doi:10.3390/foods15132374)
Supplement: Supplementary file 1 [file foods-15-02374-s001.zip › foods-4329726-supplementary.pdf]

Table S1. Fatty acid profile of pomegranate peel (percentage of the total fatty acid profile) of 'Mollar de Elche' Pomegranate: Influence of Farming system, Location and Season (2022-2023).

	2022						2023					
	**CC**	**CE**	**OC**	**OE**	**EC**	**EE**	**CC**	**CE**	**OC**	**OE**	**EC**	**EE**
Myristic acid	21.51	10.22	16.07	31.37	32.55	34.54	8.87	2.34	3.69	16.22	16.33	29.47
Palmitic acid	11.22	12.85	10.60	9.02	9.52	9.06	17.01	16.83	15.83	16.46	18.51	13.63
Stearic acid	1.69	1.98	1.36	1.35	1.27	1.29	1.79	2.08	2.62	1.87	3.57	4.67
Oleic acid	12.89	14.34	10.69	10.81	12.41	12.75	19.74	21.28	25.55	18.91	21.53	17.83
Linoleic acid	22.43	25.77	23.24	21.22	18.31	17.97	27.26	36.36	29.43	23.24	23.27	18.70
Alpha Linolenic acid	27.39	30.36	23.94	22.63	24.13	22.63	*22.97*	*19.13*	21.63	21.71	16.76	15.66
∑SFA	34.42	25.05	28.03	41.74	43.34	44.89	27.67	21.25	22.14	34.55	38.41	47.77
∑MUFA	12.89	14.34	10.69	10.81	12.41	12.75	19.74	21.28	25.55	18.91	21.53	17.83
∑PUFA	49.82	56.13	47.18	43.85	42.44	40.60	50.23	55.49	51.06	44.95	40.03	34.36

CC: Crevillente Conventional; CE: Crevillente Organic; OC: Orihuela Conventional; OE. Orihuela Organic EC: Elche Conventional; EE: Elche Organic. Myristic acid (C14:0); Palmitic acid (C16:0); Stearic acid (C18:0); Oleic acid (C18:1 c9); Linoleic acid (C18:2 c9.12); Alpha Linolenic acid (C18:3 c9.12.15 alpha.

Table S2. Fatty acid profile of pomegranate seed (percentage of the total fatty acid profile) of 'Mollar de Elche' Pomegranate: Influence of Farming system, Location and Season (2022-2023).

	2022						2023					
	**CC**	**CE**	**OC**	**OE**	**EC**	**EE**	**CC**	**CE**	**OC**	**OE**	**EC**	**EE**
Capric acid	<0.01	<0.01	<0.01	<0.01	0.01	0.01	<0.01	0.01	0.04	<0.01	0.01	0.01
Lauric acid	0.21	0.14	0.15	0.21	0.19	0.18	0.17	0.21	0.27	0.14	0.30	0.38
Myristic acid	0.02	0.01	0.03	0.03	0.02	0.02	0.03	0.04	0.17	0.03	0.04	0.06
Pentadecanoic acid	0.01	0.01	0.01	0.01	0.01	0.01	0.01	0.02	0.03	0.01	0.02	0.02
Palmitic acid	2.96	2.64	2.68	2.91	2.74	2.97	3.20	3.15	3.59	3.06	2.85	3.12
Palmitoleic acid	0.01	0.01	0.02	0.01	0.01	0.03	0.01	0.03	0.03	0.01	0.03	0.05
Margaric acid	0.03	0.02	0.03	0.03	0.02	0.02	0.04	0.04	0.05	0.04	0.04	0.05
Stearic acid	1.12	1.03	0.96	1.10	1.00	1.00	1.52	1.74	1.59	1.58	1.56	1.69
Elaidic acid	0.01	0.01	0.01	0.01	0.01	0.01	<0.01	0.01	<0.01	<0.01	0.01	0.02
Oleic acid	3.55	2.69	2.58	2.83	2.88	2.87	3.47	3.60	3.64	3.04	2.79	3.31
Vaccenic acid	0.93	0.80	0.80	0.85	0.88	0.89	0.57	0.53	0.57	0.55	0.56	0.57
Linoelaidic acid	<0.01	<0.01	<0.01	<0.01	<0.01	<0.01	<0.01	<0.01	0.01	<0.01	<0.01	<0.01
Linoleic acid	5.55	4.80	4.86	5.74	5.18	5.02	4.76	4.56	4.75	4.67	4.03	4.53
Arachidic acid	0.20	0.19	0.18	0.19	0.17	0.17	0.37	0.38	0.39	0.38	0.39	0.39
Gondoic acid	0.35	0.34	0.37	0.35	0.35	0.42	0.49	0.50	0.50	0.49	0.49	0.47
Heneicosanoic acid	0.01	0.01	0.01	0.01	0.01	0.02	0.05	0.14	0.06	0.04	0.09	0.16
Eicosadienoic acid	0.02	0.02	0.02	0.01	0.02	0.02	0.01	0.01	0.02	0.01	0.01	0.01
Behenic acid	0.07	0.07	0.06	0.07	0.07	0.07	0.17	0.18	0.19	0.14	0.18	0.18
Tricosanoic acid	0.10	0.12	0.09	0.08	0.08	0.07	0.23	0.19	0.23	0.23	0.18	0.17
Punicic acid	72.48	74.96	72.89	71.68	73.08	72.44	78.08	75.03	76.57	76.26	76.01	75.72
Lignoceric acid	9.45	9.41	10.81	10.56	10.32	10.62	5.58	7.81	6.05	7.59	8.44	7.27
Nervonic acid	0.01	<0.01	0.01	0.01	0.02	0.03	0.05	0.04	0.02	0.04	0.02	0.04
∑SFA	14.18	13.65	15.01	15.2	14.64	15.16	11.37	13.91	12.66	13.24	14.1	13.5
∑MUFA	4.86	3.85	3.79	4.06	4.15	4.25	4.59	4.71	4.76	4.13	3.9	4.46
∑PUFA	78.05	79.78	77.77	77.43	78.28	77.48	82.85	79.6	81.35	80.94	80.05	80.26

CC: Crevillente Conventional; CE: Crevillente Organic; OC: Orihuela Conventional; OE. Orihuela Organic EC: Elche Conventional; EE: Elche Organic. Saturated Fatty Acids: Capric acid (C10:0); Lauric acid (C12:0); Myristic acid (C14:0); Pentadecanoic acid (C15:0); Palmitic acid (C16:0); Margaric acid/Heptadecanoic acid (C17:0); Stearic acid (C18:0);

Arachidic acid (C20:0); Heneicosylic acid/Heneicosanoic acid/ (C21:0); Behenic acid (C22:0); Tricosylic acid/Tricosanoic acid/ (C23:0); Lignoceric acid (C24:0). Mono Unsaturated Fatty Acids: Palmitoleic acid (C16:1 c9/C16:1 n7); Elaidic acid (C18:1n9t); Oleic acid (C18:1c9/C18:1n9); Vaccenic acid (C18:1c11/C18:1n7); Gondoic acid (C20:1c11/C20:1n9); Nervonic acid (C24:1c15/C24:1n9). Polyunsaturated Fatty Acids. Linoelaidic acid (C18:2t9.12/C18:2n6t); Eicosadienoic acid (C20:2 c11.14); Linoleic acid (C18:2 c9.12/C18:2n6c); Punicic acid (C18:3-9cis. 11trans. 13cis).
